# Physiological and psychological effects of Wagyu beef taste stimulation: a randomized crossover trial

**DOI:** 10.1038/s41598-026-48123-z

**Published:** 2026-04-30

**Authors:** Harumi Ikei, Hyunju Jo, Hideki Hirano, Yoshifumi Miyazaki

**Affiliations:** 1 Institute for Advanced Academic Research, Chiba University, 1–33 Yayoi-cho, Inage-ku, Chiba, 263–8522 Japan; 2 Center for Environment, Health and Field Sciences, Chiba University, 6-2-1 Kashiwa-no-ha, Kashiwa, Chiba, 277–0882 Japan; 3 Himeji University, 2042-2 Oshio-cho, Himeji, Hyogo, Japan; 4 Hyogo Mucuna Beans Productive Cooperation, Hyogo, Japan

**Keywords:** Autonomic nervous activity, Heart rate variability, Physiological–psychological interactions, Semantic differential method, Taste stimulation, Wagyu beef, Public health, Quality of life

## Abstract

The effects of Japanese Wagyu beef consumption have primarily relied on subjective assessments, with limited investigation into physiological indicators. This study aimed to evaluate psychological and physiological responses to taste stimulation with Wagyu beef in a controlled setting. Forty-nine healthy university students participated. Psychological responses were assessed using a modified semantic differential method and the Profile of Mood States, Second Edition. Physiological responses were measured using heart rate variability to evaluate autonomic nervous activity and near-infrared time-resolved spectroscopy to assess prefrontal cortex oxyhemoglobin concentrations. The experimental stimulus comprised 4 g of Wagyu beef, whereas an equal amount of soy-based alternative meat was used as a control. Compared with the control, taste stimulation with Wagyu beef substantially enhanced subjective feelings of comfort and relaxation and improved mood states. Although no direct differences were observed in autonomic nervous activity or prefrontal cortex activity between conditions, correlation analyses revealed that higher subjective ratings of deliciousness were associated with increased parasympathetic activity during rest and reduced sympathetic activity under stress. In conclusion, these findings demonstrate that perceived deliciousness evoked by Wagyu beef is correlated with physiological relaxation, as reflected in modulation of autonomic nervous system activity.

*Trial registration*: University Hospital Medical Information Network Clinical Trials Registry (UMINCTR), UMIN000048826 (registered on 2 September 2022) and UMIN000046575 (registered on 7 January 2022).

**Supplementary Information:**

The online version contains supplementary material available at 10.1038/s41598-026-48123-z.

## Introduction

Wagyu is a Japanese beef cattle breed that is widely popular worldwide. Hence, the number of restaurants serving Japanese food, including Wagyu beef, overseas has increased annually^1^. Additionally, the export value of Japanese agricultural, forestry, and fishery products and food is predicted to reach a record high in 2024^2^, with Japanese beef, including Wagyu, accounting for around 60% of all livestock-based products^3^.

Sensory evaluation by consumers and trained panelists is the main method used to assess the taste of beef, including Wagyu beef^4^. The palatability of beef is evaluated based on various parameters such as tenderness, juiciness, and flavor, and individual preferences vary depending on factors such as country, culture, and sex^5^. Wagyu beef is rich in intramuscular fat (commonly known as marbling) and contains high levels of umami components (such as nucleic and glutamic acid), which contribute to its characteristic tenderness, juiciness, and flavor^6^. In sensory tests, Wagyu beef receives higher ratings than meat from other cattle breeds^7,8^ and is widely preferred. However, there are no reports on the effects of Wagyu beef taste stimulation on human physiological responses such as autonomic nervous activity or brain activity. Thus, further studies on this topic are warranted.

Our research group—the Nature Therapy Laboratory at Chiba University—has long investigated how exposure to natural stimuli through the five senses affects human physiological and psychological states. These studies have employed a consistent physiological measurement system, including heart rate variability (HRV) to evaluate autonomic nervous activity and near-infrared spectroscopy (NIRS) to assess prefrontal cortex activity.). Extensive data from our previous studies on olfactory^9–13^, tactile^14–20^, visual^21–25^, and auditory stimuli^26–28^ consistently demonstrate increased parasympathetic nervous activity, decreased sympathetic nervous activity, and reduced prefrontal cortex. Regarding psychological responses, we have demonstrated that natural stimuli enhance subjective feelings of comfort and relaxation, increase positive mood states, and reduce negative mood states as measured by the Profile of Mood States (POMS). A recent bibliometric analysis identified our group as the leading global contributor to scientific publications in nature therapy^29^.

Whereas sensory food evaluation has traditionally relied on subjective measures^4–8^, understanding how subjective perceptions of deliciousness relate to physiological responses is essential for advancing psychophysiology and nutrition science. Drawing on the robust physiological measurement framework established by our nature therapy research^9–28^, including reliable assessments of autonomic nervous activity and psychological indices, we aimed to integrate these approaches to explore how pleasant taste experiences influence human relaxation and well-being. Clarifying these relationships provides a scientific foundation for developing food-based interventions that promote both mental and physical health.

The current study aimed to investigate the psychological and physiological effects of Wagyu beef taste stimulation in young adults, a topic that has not been previously explored. It also sought to examine the relationship between these effects and subjective perceptions of taste. The taste stimulus was prepared from *psoas major* (Chateaubriand), a tender and palatable Wagyu beef cut. As a control, soy-based alternative meat was selected. This plant-based alternative is becoming increasingly common in Japan and worldwide and was used to compare the effects of Wagyu beef.

## Results and discussion

### Taste and odor intensity

For the subjective intensity of taste, Wagyu beef was rated as having a moderate taste (6.3 ± 0.2), whereas the control (soy-based meat) was rated as having a weak to moderate taste (5.3 ± 0.3). Hence, the two types of meat significantly differed in terms of the subjective intensity of taste (*p* = 0.003, *PS*_*dep*_ = 0.510). For the subjective intensity of odor, both Wagyu beef and the control were rated as having a weak to moderate odor (Wagyu beef: 4.9 ± 0.3; control: 4.7 ± 0.4), and no significant difference was observed between the two (*p* = 0.394).

### Psychological assessments

**Fig. 1:**
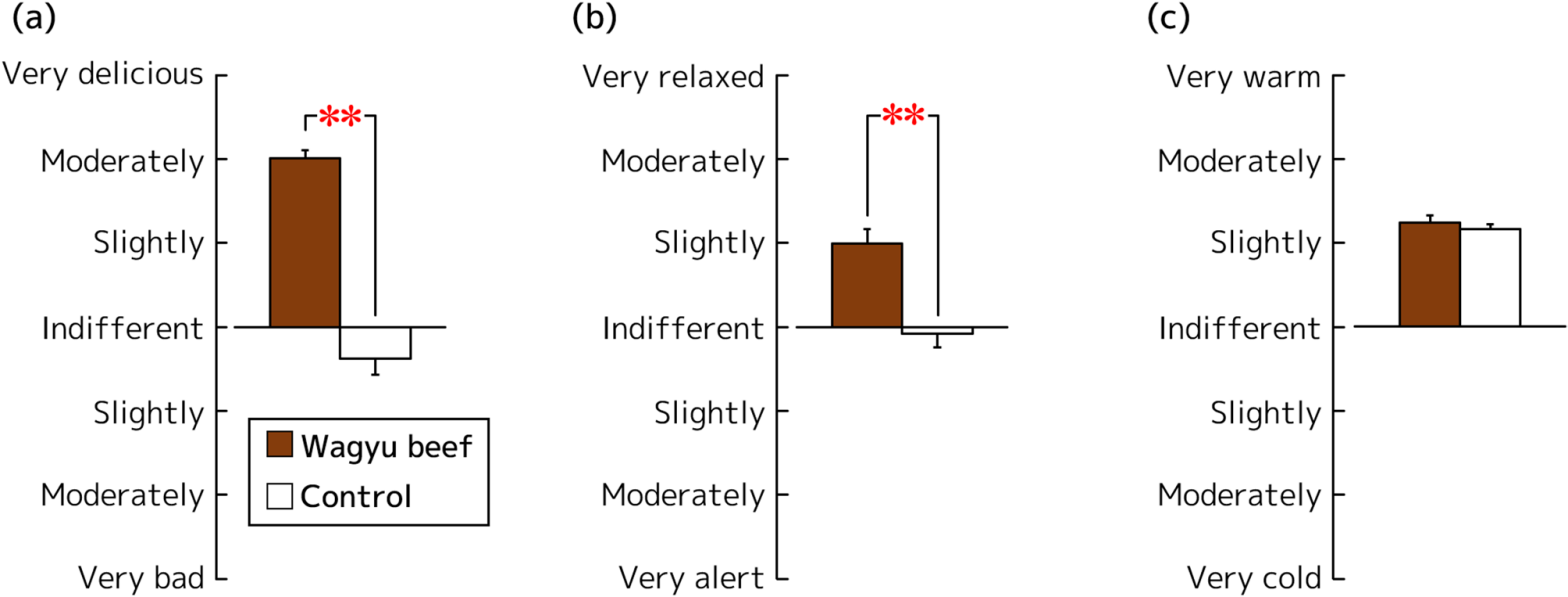
Impression evaluation after the consumption of Wagyu beef and control samples, assessed using a modified SD method. Feelings of (**a**) deliciousness, (**b**) relaxation, and (**c**) warmth are shown. Data are presented as mean ± standard error (SE). Statistical significance was determined using the Wilcoxon signed-rank test (**p < 0.01; *N* = 49).

Figure 1 shows the results of the impression evaluation using the modified SD method. For the feeling of deliciousness, Wagyu beef was rated as moderately delicious (4.0 ± 0.2) and control as between indifferent and slightly bad (− 0.8 ± 0.4). Wagyu beef was significantly more delicious than control (*p* < 0.001; Hodges–Lehmann estimated median difference = 5.0, 95% CI [4.0, 6.0]; *PS*_*dep*_ = 0.898; Fig. 1a). For relaxation, Wagyu beef was perceived as slightly relaxing (2.0 ± 0.3) and control as indifferent (− 0.2 ± 0.3), with a significant difference (*p* < 0.001; median difference = 2.5, 95% CI [1.5, 3.0]; *PS*_*dep*_ = 0.694; Fig. 1b). Warmth ratings were similar between the two (Wagyu beef: 2.5 ± 0.2; control: 2.3 ± 0.1), with no significant difference (*p* = 0.263; median difference = 0.0, 95% CI [0.0, 5.0]; Fig. 1c). Overall, Wagyu beef taste stimulation was significantly more effective than the control in enhancing perceptions of deliciousness and relaxation.

**Fig. 2:**
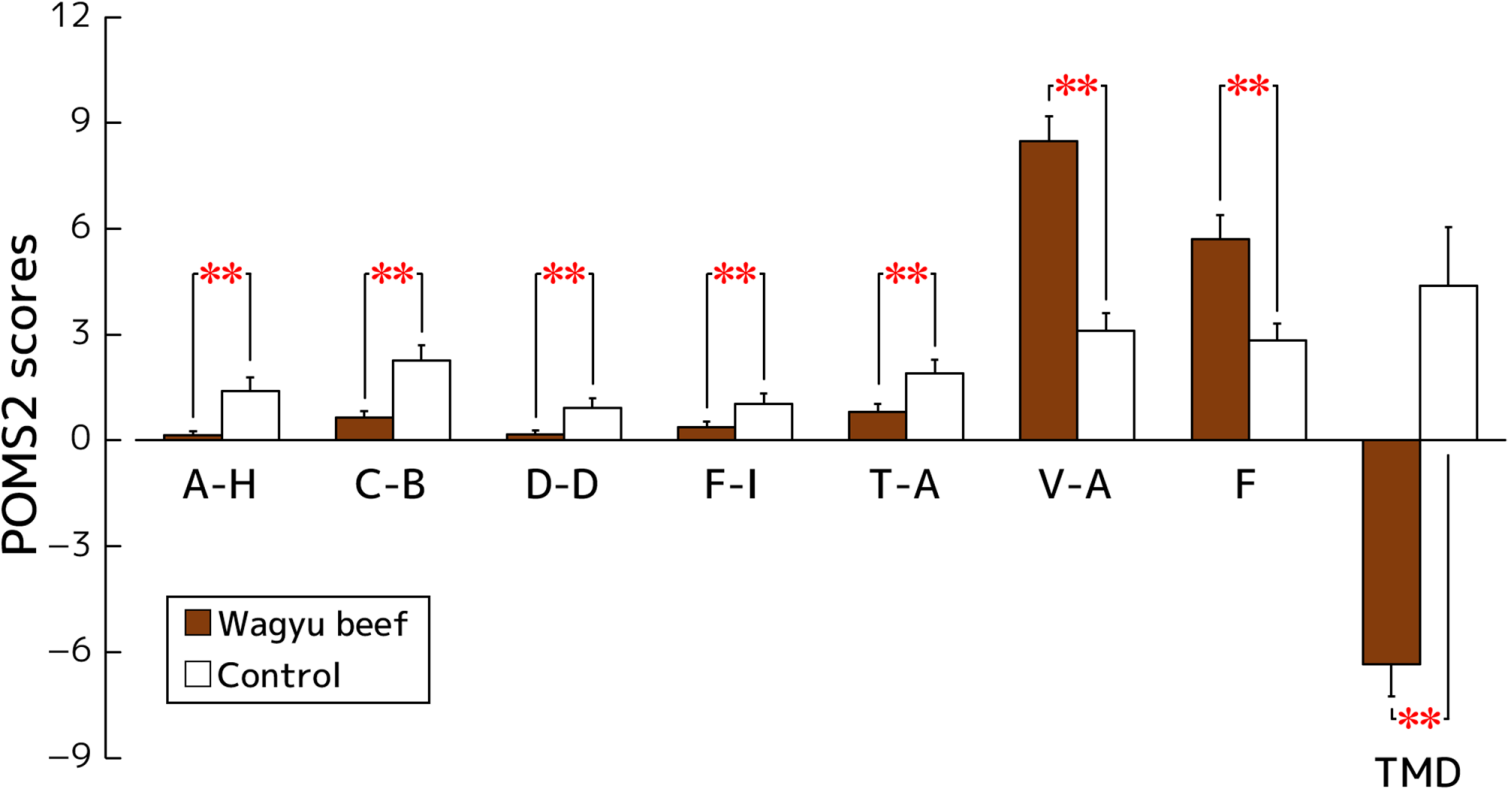
Mood evaluation based on the short version of POMS 2 following Wagyu beef and control consumption. A–H, anger–hostility; C–B, confusion–bewilderment; D–D, depression–dejection; F, friendliness; F–I, fatigue–inertia; T–A, tension–anxiety; TMD, total mood disturbance; V–A, vigor–activity. Data are presented as mean ± standard error (SE). Statistical significance was determined using the Wilcoxon signed-rank test (**p < 0.01; *N* = 49).

Figure 2 illustrates the results of the mood evaluation using the short version of the POMS 2. Negative mood subscale scores were significantly lower after tasting Wagyu beef than the control: anger–hostility (A–H: 0.1 ± 0.1 vs. 1.4 ± 0.4; *p* < 0.001; median difference = − 0.5, 95% CI [− 1.0, 0.0]; *PS*_*dep*_ = 0.408), confusion–bewilderment (C–B: 0.6 ± 0.2 vs. 2.2 ± 0.4; *p* < 0.001; median difference = − 1.0, 95% CI [− 2.0, − 0.5]; *PS*_*dep*_ = 0.490), depression–dejection (D–D: 0.2 ± 0.1 vs. 0.9 ± 0.3; *p* = 0.001; median difference = 0.0, 95% CI [− 0.5, 0.0]; *PS*_*dep*_ = 0.265), fatigue–inertia (F–I: 0.4 ± 0.2 vs. 1.0 ± 0.3; *p* = 0.004; median difference = 0.0, 95% CI [− 0.5, 0.0]; *PS*_*dep*_ = 0.286), and tension–anxiety (T–A: 0.8 ± 0.2 vs. 1.9 ± 0.4; *p* = 0.004; median difference = -0.5, 95% CI [− 1.5, 0.0]; *PS*_*dep*_ = 0.469). Positive subscale scores were significantly higher after consuming Wagyu beef: vigor–activity (V–A: 8.5 ± 0.7 vs. 3.1 ± 0.5; *p* < 0.001; median difference = 5.5, 95% CI [4.0, 7.0]; *PS*_*dep*_ = 0.837) and friendliness (F: 5.7 ± 0.7 vs. 2.8 ± 0.5; *p* < 0.001; median difference = 2.5, 95% CI [2.0, 3.5]; *PS*_*dep*_ = 0.694). The total mood disturbance score, representing overall mood status, was significantly lower after consuming Wagyu beef (− 6.4 ± 0.9) compared with the control (4.4 ± 1.7; *p* < 0.001; median difference = །8.0, 95% CI [།12.5, །6.5]; *PS*_*dep*_ = 0.837). Therefore, Wagyu beef taste stimulation had a significantly more positive impact on mood compared to the control.

### Physiological assessments

There were no significant differences in respiratory rate between conditions; thus, HRV data were analyzed. No significant difference was observed in ln(HF), a marker of parasympathetic nervous activity, between Wagyu and the control (5.65 ± 0.13 vs. 5.73 ± 0.12 lnms²; mean difference = − 0.08, 95% CI [− 0.18, 0.03]; *t*(48) = − 1.452, *p* = 0.153). Similarly, ln(LF/HF), an indicator of sympathetic nervous activity, did not differ significantly between conditions (0.63 ± 0.15 vs. 0.56 ± 0.13; mean difference = 0.06, 95% CI [− 0.14, 0.26]; *t*(48) = 0.626, *p* = 0.534).

Oxygenated hemoglobin (oxy-Hb) concentrations in the prefrontal cortex, measured using TRS—also showed no significant differences between Wagyu and the control. Left prefrontal cortex oxy-Hb concentrations were 50.7 ± 1.1µM for Wagyu and 50.8 ± 1.1 µM for the control (mean difference = − 0.15, 95% CI [− 0.62, 0.33]; *t*(48) = − 0.617, *p* = 0.540), and right prefrontal cortex levels were 52.9 ± 1.2 and 53.0 ± 1.1 µM, respectively (mean difference = − 0.12, 95% CI [− 0.70, 0.46]; *t*(48) = − 0.416, *p* = 0.679).

### Relationship between physiological and psychological changes

Using psychological indices, Wagyu beef was subjectively rated as more delicious and more relaxing than control (Fig. 1) and it promoted a better mood state (Fig. 2). However, there were no significant differences between Wagyu beef and control based on the autonomic nervous and brain activity indices of the participants.

**Fig. 3:**
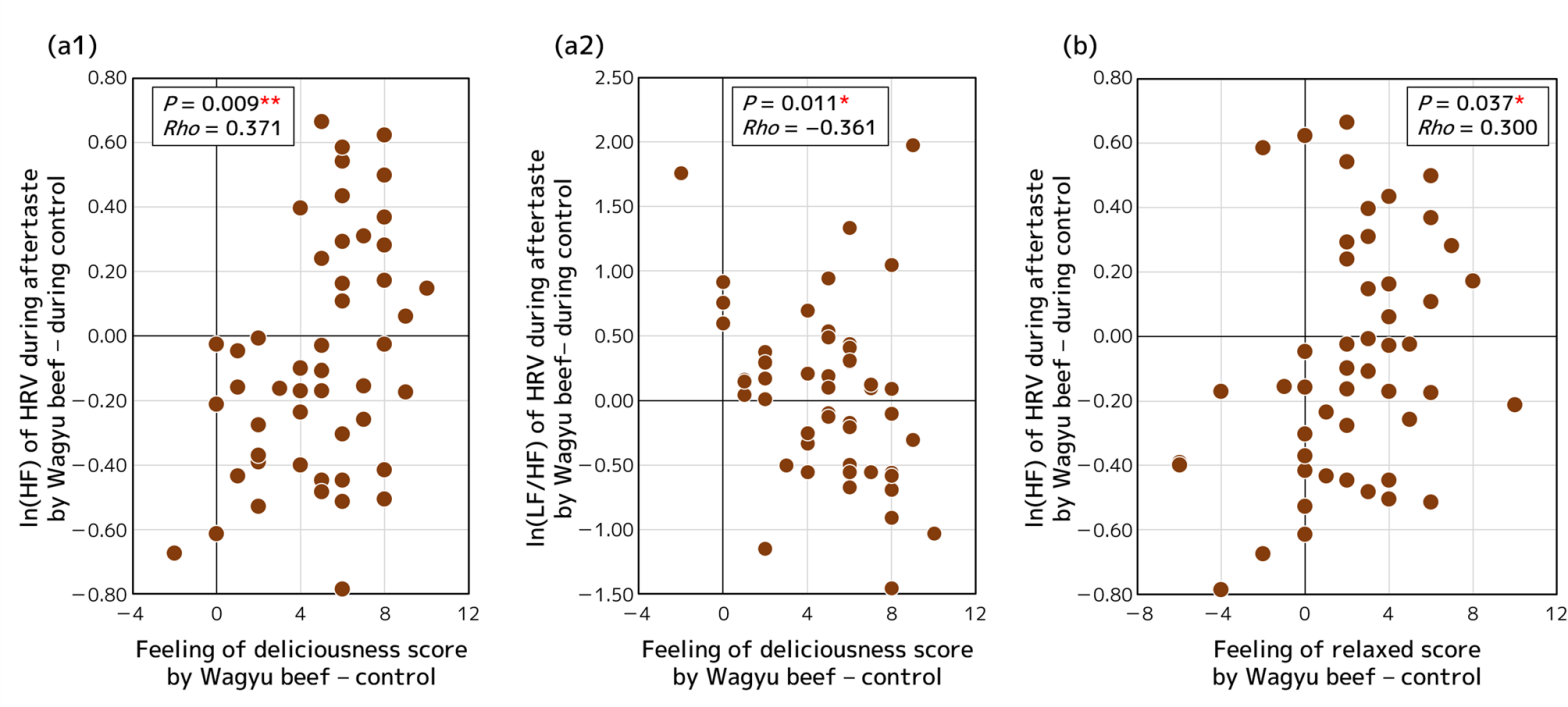
Correlation between subjective evaluations and autonomic nervous activity after taste stimulation. (**a1**) Correlation between subjective feelings of deliciousness and ln(HF) of HRV, reflecting parasympathetic nervous activity. (**a2**) Correlation between subjective feelings of deliciousness and ln(LF/HF) of HRV, reflecting sympathetic nervous activity. (**b**) Correlation between subjective feelings of relaxation and ln(HF) of HRV, which reflects parasympathetic nervous activity. Each point represents one participant (*N* = 49). Spearman’s rank correlation analysis (*Rho*) and p-values were calculated (*p < 0.05, **p < 0.01).

Next, the correlation between subjective feelings of deliciousness (minus control from Wagyu beef, Wagyu beef – control) and physiological indices (Wagyu beef –control) was examined. Results showed a significant positive correlation between subjective feelings of deliciousness and ln(HF) of HRV, an index of parasympathetic nervous activity (*p* = 0.009, *Rho* = 0.371, 95% BCa CI [0.14, 0.57]; Fig. 3-a1), and a significant negative correlation between subjective feelings of deliciousness and ln(LF/HF) of HRV, an index of sympathetic nervous activity (*p* = 0.011, *Rho* = − 0.361, 95% BCa CI [− 0.62, − 0.06]; Fig. 3-a2). In addition, significant positive correlations were found between subjective feelings of relaxation and ln(HF) (*p* = 0.037, *Rho* = 0.300, 95% BCa CI [− 0.01, 0.54]; Fig. 3-b). However, there were no significant correlations between subjective feelings of deliciousness and relaxation and brain activity indices (left and right prefrontal oxy-Hb concentrations).

Based on the analysis results, the subjective feeling of deliciousness improved with the consumption of Wagyu beef. As the sensation of deliciousness intensified, parasympathetic nervous activity, which typically increases during relaxation, also increased, while sympathetic nervous activity, which rises during stress, decreased. The associations were moderate in strength (*Rho* ≈ 0.3–0.4). These findings suggest that consuming highly palatable Wagyu beef induces physiological relaxation and exerts stress-reducing effects on the human body, particularly when the sensation of deliciousness is heightened. To our knowledge, this is the first study to examine the physiological effects of Wagyu beef taste stimulation in humans and demonstrate a significant association between subjective taste experiences and autonomic nervous system responses.

Research exploring the link between taste perception and autonomic nervous activity is still limited. Okazaki et al.^30^ reported that bitter and sour tastes elicit stronger sympathetic responses compared with sweet tastes, suggesting that physiological responses vary by the sensory properties of taste. Jiang et al.’s^31^ comprehensive review of food, eating behavior, and emotion emphasized the importance of investigating how subjective sensory experiences—particularly pleasurable sensations such as deliciousness—relate to physiological indicators such as HRV. The present study addresses this research gap by showing that higher subjective ratings of deliciousness from Wagyu beef consumption are significantly associated with enhanced parasympathetic activity and reduced sympathetic activity. This suggests that positive emotional experiences related to taste may foster physiological relaxation.

Moreover, this study extends previous research on psychological and physiological responses to natural sensory stimuli, which have traditionally been examined with olfactory, tactile, visual, and auditory modalities in the field of nature therapy^9–28^. These studies consistently demonstrate that pleasant natural stimuli activate the parasympathetic nervous system and reduce sympathetic activity, promoting relaxation and stress recovery. Our findings suggest that taste may serve as an additional sensory pathway capable of eliciting similar psychophysiological responses, offering a complementary perspective to existing nature therapy research.

Our findings also contribute to the broader understanding of how subjective emotional experiences related to food can influence physiological states. By integrating validated physiological measures of autonomic nervous activity with psychological evaluations of taste perception, this study bridges the fields of psychophysiology and nutritional science. The results provide preliminary evidence that pleasurable taste experiences are associated with changes in autonomic indices, thereby offering a foundation for further investigation of taste-related stimuli in stress-related contexts.

## Limitations

This study is the first to investigate the relationship between subjective evaluations and physiological responses during beef taste stimulation. The following limitations should be acknowledged:

1. The Wagyu beef samples were obtained from two carcasses to prioritize consistent quality and experimental control, which was essential for this initial exploratory study of physiological responses. However, the limited number of carcasses restricts generalizability. Future studies involving a larger number of carcasses and different beef grades are required to enhance the robustness and external validity of these findings.
2. Very few studies have evaluated the physiological effects related to taste stimulation. As an initial exploratory study, a soy-based alternative was used as the control. In future studies, comparisons with ordinary beef are necessary to clarify the specific effects of Wagyu beef taste stimulation.
3. Although compositional analyses of the major components were conducted for both Wagyu beef and the soy-based alternative (Supplementary Tables S1–S3), pH and other taste-related compounds were not analyzed. Future studies should include more comprehensive compositional profiling to confirm sample representativeness and reproducibility.
4. The participants were Japanese men and women in their twenties. Cultural familiarity with Wagyu beef may have influenced the results. Future studies should include participants of different nationalities and age groups to ensure broader generalizability.
5. Previous studies on the psychological and physiological effects of sensory stimuli have typically compared group-level means. This study examined individual-level correlations between psychological and physiological changes. Future research should expand on this approach to explore person-level variability.
6. This study focused on acute taste stimulation. Large-scale analyses by Dobersek et al.^32^ suggested that habitual meat consumption may be associated with lower depression and anxiety levels. Future research should investigate the long-term effects of beef consumption, including Wagyu, on physiological and psychological well-being.

In light of these limitations, future research will focus on two complementary directions that directly extend the present findings (Limitations 5 and 6). First, the person-level analytic framework employed here will be expanded to further characterize individual variability and assess its robustness across additional sensory modalities, including olfactory, visual, and tactile stimuli. Second, longitudinal or repeated-exposure designs will be required to evaluate sustained physiological and psychological responses associated with habitual beef consumption, including Wagyu. In parallel, and in subsequent studies, remaining sources of limited generalizability (Limitations 1–4) will be addressed by increasing the number of carcasses, comparing different beef types and grades, expanding compositional profiling (including pH and taste-related compounds), and recruiting more diverse participant populations across age groups and cultural backgrounds. Such research will be important for building a stronger empirical foundation to better understand psychophysiological processes related to stress and well-being.

## Conclusion

This study suggests that taste stimulation with Wagyu beef induces physiological relaxation as the perception of deliciousness increases. Specifically, the subjective perception of deliciousness was moderately associated with increased parasympathetic nervous activity and decreased sympathetic activity. Therefore, the emotional experience of palatability may influence autonomic nervous function. By examining taste as a sensory modality, this study extends nature-therapy research by evaluating associations between subjective taste perception and autonomic responses.

## Materials and methods

### Samples

Wagyu beef was sourced from two Japanese Black cattle heifers (Tajima strain) raised in Hyogo Prefecture, Japan, and supplied by Ueda Chikusan Co., Ltd. (Hyogo, Japan). The beef samples were obtained from two carcasses to ensure consistent quality and experimental control. Based on their individual identification numbers (15098-6645-6 and 15098-5195-7), the exact ages at slaughter were 33 and 38 months, respectively. This strain, commonly referred to as Tajima-gyu, is a representative line of Wagyu beef selectively bred for over 100 years and strongly influences all existing Japanese Black cattle breeds^6^.

For the experiment, the *psoas major* (Chateaubriand), a highly tender and palatable cut of Wagyu beef, was selected. This cut was chosen because the purpose of the study was to evaluate the physiological and psychological responses induced by taste stimulation perceived as delicious. The psoas major muscles were sampled from the carcasses after postmortem aging at 0 °C under hygienic conditions at the supplier’s facility (21 days for identification number 15098-5195-7 and 31 days for identification number 15098-6645-6). After aging, the meat was cut into bite-sized pieces, vacuum-sealed, and stored at − 18 °C until use. Samples were thawed in a refrigerator at 5 °C the day before testing.

The control meat was a commercially available, soy-based alternative (retort-type), produced by a Japanese manufacturer, with whole soybeans as the main ingredient. Chicken breast was used as a dummy stimulus during the pre-experiment practice to familiarize participants with the measurement procedure and was excluded from the primary analysis. The control and chicken breast were stored and thawed under the same conditions as the Wagyu beef. Note, owing to the obvious sensory differences between the two samples, neither participants nor experimenters were blinded to the intervention condition.

On the experiment day, each type of meat was cut into approximately 4 g portions (Wagyu beef: 4.01 ± 0.09 g; control: 4.00 ± 0.11 g; chicken: 3.99 ± 0.25 g). The size of each piece of Wagyu beef was approximately 2 × 2 cm. Each piece was placed on a microwave-safe plastic tray, wrapped in plastic, and refrigerated until use. Before serving, the meat was heated in a microwave at 800 W for 20 s and allowed to cool at room temperature (~ 20 °C) for 6 min, reaching an internal temperature of approximately 35 °C. No seasonings were added.

Participants rated the taste and smell intensity of each sample using an 11-point scale: 0 = no taste/smell, 2 = slight, 4 = weak, 6 = *moderate*, 8 = *strong*, and 10 = unbearably strong.

### Participants

Participants were healthy Japanese undergraduate and graduate students (28 men, 21 women; total *N* = 49). Only individuals in good physical and mental health were included. Those who had food allergies, chronic respiratory diseases like asthma or rhinitis, or any chronic medical or psychiatric disorders were excluded. Participants receiving medical treatment for any condition, including hormone replacement therapy, pharmacotherapy, exercise therapy, or dietary therapy, were also excluded. Individuals who habitually smoked, had cardiac arrhythmia, or a tendency to become drowsy during experimental sessions were not eligible. Female participants were excluded if they were menstruating on the day of the experiment. All participants were instructed to avoid strenuous exercise, alcohol consumption, and sleep deprivation on the day before the experiment. On the day of the experiment, participants were asked to refrain from consuming caffeinated beverages, such as coffee or tea, as well as strongly flavored or spicy foods that might affect their taste perception. They were also instructed to complete their last meal at least one hour before the session.

Sample size estimation was conducted using G*Power 3.1.9.7^33,34^ under three statistical scenarios: Wilcoxon signed-rank test (subjective parameters; two-tailed; effect size = 0.5; α = 0.05; power = 0.80); paired t test (physiological indicators; two-tailed; effect size = 0.5; α = 0.05; power = 0.80); and Spearman’s rank correlation analysis (subjective–physiological correlation; since G*Power 3.1.9.7 does not include a dedicated test for Spearman’s correlation, we followed common practice and used the correlation module for Pearson’s correlation with equivalent parameters: two-tailed; effect size = 0.4; α = 0.05; power = 0.80). As a result, the required sample sizes were 35, 34, and 44, respectively.

To account for potential dropouts (~ 15%), a target of 52 participants was set (44/0.85). Initially, 55 participants (32 men and 23 women) were recruited; however, six dropped out because of illness or other reasons, resulting in a final sample of 49. Table 1 shows the physical characteristics of the participants.

**Table 1:** Physical characteristics of the participants.

Parameters	All participants (*N* = 49)	Male participants (*N* = 28)	Female participants (*N* = 21)
Age (years)	21.8 ± 2.0	21.9 ± 2.2	21.6 ± 1.8
Height (cm)	165.8 ± 7.0	170.5 ± 4.2	159.4 ± 4.4
Weight (kg)	58.7 ± 10.6	63.7 ± 9.6	51.9 ± 8.0
Body mass index (kg/m^2^)	21.3 ± 3.1	21.9 ± 3.0	20.4 ± 3.0

Note. Values are presented as mean ± standard deviation (SD). BMI categories were defined according to the World Health Organization (WHO) classification: underweight (< 18.5), normal (18.5–24.9), and overweight (≥ 25.0). The mean BMI of each group was within the normal range overall. Among all participants, nine were underweight (two males and seven females) and three were overweight (two males and one female).

#### Ethical approval

was granted by the Ethics Committee of the Center for Environment, Health and Field Sciences, Chiba University (approval no. 54) and conducted in accordance with the latest Declaration of Helsinki. This study was prospectively registered with the University Hospital Medical Information Network Clinical Trials Registry (UMIN-CTR) under two separate registration numbers according to participant sex: UMIN000048826 for male participants (registered on 2 September 2022) and UMIN000046575 for female participants (registered on 7 January 2022). Male and female participants were recruited and tested under their respective trial registrations. All participants gave written informed consent after being briefed on the study.

### Study protocol

**Fig. 4:**
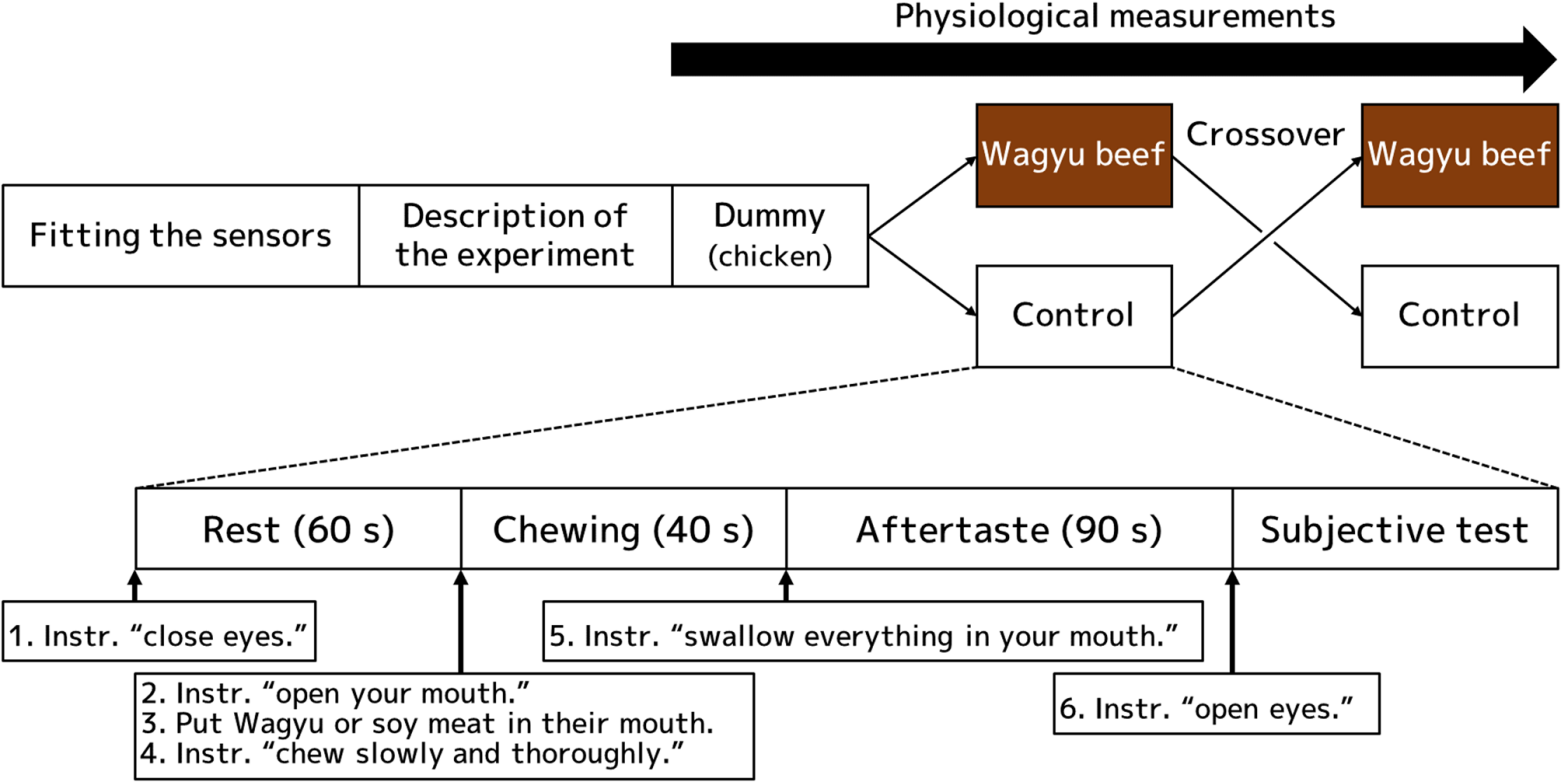
Experimental procedure. Instr.: instruction.

The intervention was a taste stimulus, and a one-factor, two-condition (Wagyu beef and control), crossover comparison study (within-subject experiment) was conducted (Fig. 4). H.I. generated the allocation sequence and assigned participants to one of the two tasting orders (Wagyu beef first or control first) using stratified block randomization, with sex as the stratification factor. The experiment was conducted in a soundproof artificial climate chamber maintained at 24 °C and 50% relative humidity. Participants entered the chamber individually, and physiological sensors were attached to the forehead and chest.

Before the main experiment, participants underwent a practice session using a dummy sample (chicken breast) following the same procedure. In the actual experiment, each session began with 60 s of rest in a seated position with eyes closed, the participants opened their mouths based on the cue of the experimenter. Then, they ingested the beef or control and chewed slowly for 40 s. After swallowing based on the experimenter’s cue, the participants rested for 90 s with their eyes closed, and the physiological effects of the aftertaste were examined. Subsequently, participants opened their eyes at the experimenter’s instruction, completed a subjective evaluation questionnaire, drank water, and took a 10-min break. The second trial followed the same procedure but with the different meat sample. No adverse events or unintended harms were observed or reported during the study.

### Psychological and physiological measurements

Psychological and physiological responses were evaluated using a validated system previously employed in studies on nature-derived sensory stimuli (e.g., olfactory, tactile, visual, and auditory) and their effects on human comfort and relaxation^9–28^. To avoid redundancy and concerns regarding self-plagiarism, detailed descriptions of the measurement procedures are provided in the Appendix.

Autonomic nervous activity was assessed via HRV, specifically the natural logarithm of high-frequency components (ln[HF]) and the low-frequency-to-high-frequency ratio (ln[LF/HF])^35,36^. Simultaneously, brain activity was evaluated by measuring oxyhemoglobin (oxy-Hb) concentrations in the left and right prefrontal cortices using near-infrared time-resolved spectroscopy^37^.

Following the physiological measurements, participants completed subjective assessments of deliciousness and relaxation using a modified semantic differential (SD) method^38^, along with mood evaluations via the short version of the POMS 2^39,40^.

### Statistical analysis

All final analyses were conducted on the 49 participants who completed both experimental conditions and provided evaluable data for the outcome measures. Subjective evaluation and physiological data were expressed as mean ± standard error. A significance threshold of *p* < 0.05 was applied to all statistical tests. The Wilcoxon signed-rank test assessed differences in subjective parameters between the Wagyu beef and control conditions, whereas paired *t* tests were used for physiological indicators. 95% confidence intervals (95% CIs) were reported for all key comparisons. For nonparametric tests, Hodges–Lehmann estimated median differences and their 95% CIs were provided. Effect sizes were reported when notable differences were observed. For subjective parameters, the probability of superiority for dependent samples (*PS*_*dep*_) was calculated^41^; for physiological indicators, Cohen’s d was used^42^.

Spearman’s rank correlation analysis was conducted to explore associations between subjective and physiological measures, with the strength of associations interpreted via Spearman’s rank correlation analysis (*Rho*). 95%CIs for *Rho* were calculated using the bias-corrected and accelerated bootstrap method with 2,000 resamples.

All statistical analyses were performed using SPSS Statistics software (version 29.0; IBM Corp., Armonk, New York).

## Supplementary Information

Below is the link to the electronic supplementary material.

Supplementary Material 1

## Data Availability

The data supporting the findings of this study are available from the corresponding author upon reasonable request.
