# Supplementary material for: Physiological and psychological effects of Wagyu beef taste stimulation: a randomized crossover trial
Source: Sci Rep. 2026 Apr 30;16:20099. doi: 10.1038/s41598-026-48123-z (PMC13324031; doi:10.1038/s41598-026-48123-z)
Supplement: Supplementary file 1 — Supplementary Material 1 [file 41598_2026_48123_MOESM1_ESM.docx]

**Supplementary Materials and Methods**

Physiological and Psychological Effects of Wagyu Beef Taste Stimulation: A Randomized Crossover Trial

Harumi Ikei^1,2,5^, Hyunju Jo^2^, Hideki Hirano^3,4^, Yoshifumi Miyazaki^2,5^

^1^Institute for Advanced Academic Research, Chiba University, 1–33 Yayoi-cho, Inage-ku, Chiba 263–8522, Japan.

^2^Center for Environment, Health and Field Sciences, Chiba University, 6-2-1 Kashiwa-no-ha, Kashiwa, Chiba 277–0882, Japan.

^3^Himeji University, 2042-2 Oshio-cho, Himeji, Hyogo, Japan. 4Hyogo Mucuna Beans Productive Cooperation, Hyogo, Japan.

^5^Harumi Ikei and Yoshifumi Miyazaki contributed equally to this work. email: hikei@chiba-u.jp; ymiyazaki@faculty.chiba-u.jp

*Investigation of meat quality and ingredients*

The Wagyu beef and soy-based alternative meat’s moisture and fat content, which affect quality, 5'-inosinic acid content, which is one of the umami components [1,2], free amino acid content, and fatty acid composition were analyzed. The analysis was commissioned to the Japan Food Research Center, a general incorporated foundation. The meat was cooked using the same procedure as in the subject experiment, vacuum packed, frozen, and sent to the analysis company via frozen delivery. Supplementary Tables S1–S3 show the results.

**Supplementary Table S1.** Moisture, fat, inosine 5'-monophosphate (IMP) contents (g/100 g matter) in cooked Wagyu beef and control (alternative meat)

|  | **Wagyu beef** | **Control** |
| --- | --- | --- |
| Moisture^a^ | 48.5 | 58.1 |
| Fat^b^ | 18.1 | 0.9 |
| IMP^c^ | 0.04 | nd |

Abbreviation: nd, not detected.

ᵃAtmospheric heating drying method.

ᵇAcid hydrolysis method.

ᶜHigh-performance liquid chromatography (measured after extraction with 5% perchloric acid).

**Supplementary Table S2.** Fatty acid composition (%) of cooked Wagyu beef and control

|  | **Wagyu beef** | **Control** |
| --- | --- | --- |
| C14:0 | 1.6 | 0.1 |
| C14:1 | 0.5 | nd |
| C15:0 | 0.2 | nd |
| Iso-C16:0 | 0.1 | nd |
| C16:0 | 21.6 | 21.5 |
| C16:1 | 3.0 | 0.2 |
| Anteiso-C17:0 | 0.5 | nd |
| C17:0 | 0.6 | 0.2 |
| C17:1 | 0.6 | nd |
| C18:0 | 11.8 | 3.6 |
| C18:1 | 50.5 | 17.5 |
| C18:2n-6 | 4.2 | 49.9 |
| C18:3n-3 | 0.2 | 4.9 |
| C20:0 | nd | 0.2 |
| C20:1 | 0.3 | 0.1 |
| C20:3n-6 | 0.2 | nd |
| C20:4n-6 | 0.6 | nd |
| C22:0 | nd | 0.3 |
| C24:0 | nd | 0.2 |
| Unidentified | 3.4 | 1.3 |

*Note*. Analysis method: gas chromatography.

Abbreviation: nd, not detected.

**Supplementary Table S3.** Free amino acid contents (g/100 g matter) of cooked Wagyu beef and control

|  | **Wagyu beef** | **Control** |
| --- | --- | --- |
| Arginine | 20 | 42 |
| Lysine | 23 | 9 |
| Histidine | 9 | 5 |
| Phenylalanine | 24 | 8 |
| Tyrosine | 19 | 9 |
| Leucine | 35 | 7 |
| Isoleucine | 20 | 4 |
| Methionine | 17 | 2 |
| Valine | 23 | 4 |
| Alanine | 55 | 12 |
| Glycine | 13 | 3 |
| Proline | 5 | 5 |
| Glutamic acid | 35 | 25 |
| Serine | 22 | 4 |
| Threonine | 15 | 3 |
| Aspartic acid | 4 | 18 |
| Tryptophan | 4 | 5 |

*Note*. Analysis method: amino acid analysis. High-performance liquid chromatography was used for free tryptophan only.

*Psychological measurements*

The modified semantic differential (SD) method [3] and the short version of the Profile of Mood States Second Edition (POMS 2) [4,5] were used to evaluate the psychological effects of Wagyu beef and control (alternative meat) based on taste stimulation.

In the modified SD methods, the following three pairs of adjectives were assessed: deliciousness–bad, relaxed–alert, and warm–cold. The participants’ responses were scored on a 13-point scale, ranging from very delicious/relaxed/warm (6) to very bad/alert/cold (−6).

The POMS 2 questionnaire was used to evaluate the mood status based on the tension–anxiety (T–A), depression–dejection (D–D), anger–hostility (A–H), fatigue–inertia (F–I), confusion–bewilderment (C–B), vigor–activity (VA), and friendliness (F) scores. The participants’ responses were scored on a 5-point scale, ranging from extremely (4) to not at all (0). In addition, the total mood disturbance (TMD) score was calculated using the following formula: [(T–A) + (D–D) + (A–H) + (F–I) + (C–B) − (V–A)]. A low TMD score indicated a positive mood status.

*Physiological measurements*

Heart rate variability (HRV) was used as an index of parasympathetic and sympathetic nervous activities. HRV was analyzed during the periods between consecutive R waves (R–R intervals), as measured using a portable electrocardiograph (Activtracer AC-301A; GMS, Tokyo, Japan) [6,7]. The high-frequency (HF, 0.15–0.40 Hz) and low-frequency (LF, 0.04–0.15 Hz) power level components of HRV were calculated using the maximum entropy method (MemCalc/Win; GMS, Tokyo, Japan) [8,9]. The HF power indicated the parasympathetic nervous activity. Meanwhile, the LF/HF power ratio represented the sympathetic nervous activity [6,10]. The natural logarithmic values of HF (ln(HF)) and LE/HF (ln(LF/HF)) were used to normalize the HRV parameters among the participants [11]. Further, respiratory changes can influence HRV data. Thus, the participants’ respiratory rates were examined during the period between the two stimuli. The respiratory rate can be estimated from the HRV power spectrum [12].

Near-infrared time-resolved spectroscopy, which is a type of near-infrared spectroscopy, was used as an index for assessing the prefrontal cortex activity [13-15]. Sensors were mounted on the participant’s forehead (positions Fp1 and Fp2 in the International 10–20 system), and the oxygenated hemoglobin concentrations in the prefrontal cortex were measured using the TRS-20 system (Hamamatsu Photonics K.K., Shizuoka, Japan). All data were transformed via linear interpolation to every 1 s.

The physiological effects of Wagyu beef and control based on taste stimulation were evaluated using an average value of 90 s during aftertaste measurement.

**References**

1. Kodama, S. On a procedure for separating inosinic acid [in Japanese]. *J. Tokyo Chem. Soc*., **34**, 751-757 (1913).
2. Maga, J.A. Umami flavour of meat in Flavor of Meat and Meat Products (ed. Shahidi, F.) Springer, Boston, MA. 10.1007/978-1-4615-2177-8_6 (1994).
3. Osgood, C. E., Suci, G. J., & Tannenbaum, P. The measurement of meaning. (University of Illinois Press, 1957).
4. Heuchert, J. P., & McNair, D. M. Profile of Mood States 2. (Multi-Health Systems Inc., 2012).
5. Heuchert, J. P., & McNair, D. M. Japanese translation of POMS2: Profile of mood states. (eds. Yokoyama, K., & Watanabe, K) (Kaneko Shobo, 2015) [in Japanese].
6. Task Force of the European Society of Cardiology, the North American Society of Pacing and Electrophysiology. Heart rate variability: standards of measurement, physiological interpretation and clinical use. *Circulation*, **93**, 1043-1065; 10.1161/01.CIR.93.5.1043 (1996).
7. Kobayashi, H., Ishibashi, K., & Noguchi, H. Heart rate variability; an index for monitoring and analyzing human autonomic activities. *Appl. Human. Sci.*, **18**, 53-59; 10.2114/jpa.18.53 (1999).
8. Kanaya, N., et al. Differential effects of propofol and sevoflurane on heart rate variability. *Anesthesiology* 98, 34-40; 10.1097/00000542-200301000-00009 (2003).
9. Sawada, Y. et al. New technique for time series analysis combining the maximum entropy method and non-linear least squares method: its value in heart rate variability analysis. *Med. Biol. Eng. Comput.* **35**, 318-322; 10.1007/BF02534083 (1997).
10. Pagani, M., et al., Power spectral analysis of heart rate and arterial pressure variabilities as a marker of sympatho-vagal interaction in man and conscious dog. *Circ. Res.*, **59**, 178-193; 10.1161/01.res.59.2.178 (1986).
11. Kobayashi, H., Park, B. J., & Miyazaki, Y. Normative references of heart rate variability and salivary alpha-amylase in a healthy young male population. *J. Physiol. Anthropol.* **31**, 9; 10.1186/1880-6805-31-9 (2012).
12. Schäfer, A., & Kratky, K. W. Estimation of breathing rate from respiratory sinus arrhythmia: comparison of various methods. *Ann. Biomed. Eng.* **36**, 476-485; 10.1007/s10439-007-9428-1 (2008).
13. Torricelli, A., et al. Time domain functional NIRS imaging for human brain mapping. *Neuroimag*. **85**, 28-50; 10.1016/j.neuroimage.2013.05.106 (2014).
14. Ohmae, E., et al. Clinical evaluation of time-resolved spectroscopy by measuring cerebral hemodynamics during cardiopulmonary bypass surgery. *J. Biomed. Opt.* **12**, 062112; 10.1117/1.2804931 (2007).
15. Ohmae, E. et al. Cerebral hemodynamics evaluation by near-infrared time-resolved spectroscopy: correlation with simultaneous positron emission tomography measurements. *Neuroimage*, **29**, 697-705; 10.1016/j.neuroimage.2005.08.008 (2006).
